# Filamin A Orchestrates Cytoskeletal Structure, Cell Migration and Stem Cell Characteristics in Human Seminoma TCam-2 Cells

**DOI:** 10.3390/cells9122563

**Published:** 2020-11-30

**Authors:** Harald Welter, Carola Herrmann, Thomas Fröhlich, Florian Flenkenthaler, Katja Eubler, Hubert Schorle, Daniel Nettersheim, Artur Mayerhofer, Annette Müller-Taubenberger

**Affiliations:** 1 Anatomy III, Cell Biology, Biomedical Center, Ludwig Maximillian University of Munich, 82152 Planegg, Martinsried, Germany; welter@bmc.med.lmu.de (H.W.); carola.herrmann@bmc.med.lmu.de (C.H.); katja.eubler@bmc.med.lmu.de (K.E.); amueller@bmc.med.lmu.de (A.M.-T.); 2 Laboratory for Functional Genome Analysis (LAFUGA), Gene Center, Ludwig Maximilian University of Munich, 81377 Munich, Germany; frohlich@genzentrum.lmu.de (T.F.); flenkenthaler@genzentrum.lmu.de (F.F.); 3 Department of Developmental Pathology, Institute of Pathology, University Hospital Bonn, 53127 Bonn, Germany; schorle@uni-bonn.de; 4 Department of Urology, Urological Research Lab, Translational UroOncology, University Hospital Düsseldorf, Heinrich Heine University Düsseldorf, 40225 Düsseldorf, Germany; daniel.nettersheim@med.uni-duesseldorf.de

**Keywords:** cytoskeleton, filamin, human testis, spermatogonia, stem cell

## Abstract

Filamins are large dimeric F-actin cross-linking proteins, crucial for the mechanosensitive properties of a number of cell types. Due to their interaction with a variety of different proteins, they exert important regulatory functions. However, in the human testis the role of filamins has been insufficiently explored. Immunohistochemical staining of human testis samples identified filamin A (FLNA) in spermatogonia and peritubular myoid cells. Investigation of different testicular tumor samples indicated that seminoma also express FLNA. Moreover, mass spectrometric analyses identified FLNA as one of the most abundant proteins in human seminoma TCam-2 cells. We therefore focused on FLNA in TCam-2 cells, and identified by co-immunoprecipitation LAD1, RUVBL1 and DAZAP1, in addition to several cytoskeletal proteins, as interactors of FLNA. To study the role of FLNA in TCam-2 cells, we generated FLNA-deficient cells using the CRISPR/Cas9 system. Loss of FLNA causes an irregular arrangement of the actin cytoskeleton and mechanical instability, impaired adhesive properties and disturbed migratory behavior. Furthermore, transcriptional activity of typical stem cell factors is increased in the absence of FLNA. In summary, our data suggest that FLNA is crucially involved in balancing stem cell characteristics and invasive properties in human seminoma cells and possibly human testicular germ cells.

## 1. Introduction

Filamins (FLNs) are among the best investigated actin-binding proteins, and widely distributed in vertebrate and non-vertebrate cells [1,2,3]. The human FLN family consists of three isoforms: FLNA, FLNB and FLNC. FLNA and FLNB are ubiquitously expressed, while expression of FLNC is more specific to skeletal and cardiac muscle [4]. However, low expression of FLNC was also reported for some non-muscle cells [5].

In general, all FLNs display a similar structure, consisting of an N-terminal actin-binding domain, which contains two calponin homology domains, followed by a flexible rod segment composed of 24 immunoglobulin-like (Ig-like) repeats, and two flexible hinge domains between repeats 15 and 16 (hinge 1, H1) and repeats 23 and 24 (hinge 2, H2). The hinge regions are highly flexible regions, and were shown to contain cleavage sites for the protease calpain [2,6]. The last Ig-like repeat 24 mediates dimerization of the FLN molecule and thus is responsible for the typical V-shaped form of the dimeric protein [6]. FLNs have been mostly described as homodimers, however some studies also reported heterodimeric FLNs [7]. All three FLN isoforms share a high sequence similarity and show, in some cell types, comparable expression patterns [8], implying that FLN isoforms share overlapping functions or can compensate for each other.

It was proposed that the unfolding of FLNA may play a role in mechanoprotection and may lead to the dissociation or association of diverse binding partners from or to the protein. FLNs interact with a variety of other proteins, and thus serve as scaffolds with implicated regulatory functions [2,8]. The first identified and most important interaction partner of FLNA is actin. A large number of biochemical and structural studies explored this interaction in detail and confirmed that FLNA is a classical actin-crosslinking protein. Currently, the interactome of FLNA lists more than 350 different proteins on Biogrid [9], including transmembrane receptors, signaling molecules and transcription factors. Thus, in addition to functions of FLNA important for mechanical properties and adhesion and migration, these findings suggested that FLNA acts as a scaffolding hub and organizes various signaling pathways and other cellular processes including cell differentiation, morphogenesis and transcriptional regulation [4,8].

In humans, null or specific missense mutations of *FLNA* are associated with a wide range of genetic disorders, such as periventricular nodular heterotopia (PVHD), a brain malformation characterized by disrupted neuronal migration [10], or otopalatodigital (OPD) spectrum disorders. OPD disorders are characterized by congenital malformations including skeletal dysplasia, central nervous system defects and anomalies regarding the craniofacial, cardiac, genitourinary and intestinal system [11,12,13]. Several studies revealed that FLNA expression supports oncogenic diseases in humans such as melanoma, lung and hepatocellular cancer [14,15,16], whereas FLNA protein was decreased in breast cancer. Further, knockdown of FLNA promoted cell migration and invasion [17]. Hence, the roles of FLNA in human malignancies remain somewhat controversial.

In the present study, we have used an integrated approach to analyze the role of the most abundant FLN isoform, FLNA, in the male reproductive system. The investigation was initiated as a consequence of our original observation that FLNs are rather abundant proteins in certain cells of the human testis. We found that FLNA and FLNB are enriched in spermatogonial cells and also in certain testicular germ cell tumors. To explore the role of FLNA in more detail, we concentrated on TCam-2 cells, a model cell line derived from human seminomas [18,19,20]. These cells are considered to represent human male germ cells at an early stage of prenatal development. Our results suggest that in TCam-2 cells, FLNA is crucial not only for cellular integrity, adhesive and migratory behavior but also for specific signaling functions that are responsible for balancing pluripotency states of male germ cells.

## 2. Materials and Methods

### 2.1. Cell Culture of TCam-2 Cells and Human Testicular Biopsies

TCam-2 cells (source: Hubert Schorle, Institute of Pathology, Bonn, Germany) [18,19,20] were cultured in RPMI1640 with phenol red and L-glutamine (Biochrom, Berlin, Germany) supplemented with 10% fetal calf serum (FCS) (Capricorn Scientific, Ebsdorfergrund, Germany) plus 1% penicillin/streptomycin (Biochrom, Berlin, Germany) as previously described [21]. Testicular biopsies for immunohistochemistry were obtained from 36–55-year-old men with obstructive azoospermia but normal spermatogenesis as described in [22,23]. The study was approved by the local Ethics Committee (Technical University of Munich, Faculty of Medicine; project 491/18S-KK), and scientific use of the biopsies was permitted by written informed consent from all of the patients. The experiments were carried out in accordance with the relevant guidelines and regulations.

### 2.2. Assessment of Cell Number, Cell Size and Viability

Cell integrity, cell number and cell diameter were determined by an automated cell counting device (CASY system, Omni Life Science, Bremen, Germany). ATP content correlates with cell number and/or viability, hence the firefly luciferase assay, using a CellTiter-Glo^®^ Assay kit (Promega, Mannheim, Germany), was used to characterize TCam-2 wildtype (WT) versus FLNA-deficient cells. Luminescence was measured in a luminometer (BMG Labtech, Ortenberg, Germany).

### 2.3. Generation of FLNA-Deficient Cells by CRISPR/Cas9 Technology

Analysis for putative CRISPR targets, including prediction of off-target sites, was performed using the CRISPR design tool (http://crispr.mit.edu/). To compromise FLNA expression, TCam-2 cells were transfected simultaneously with the pX330 vector encoding selected guide RNAs (gRNA) directed towards the FLNA coding region using FuGeneHD (Promega, Mannheim, Germany), and a plasmid encoding GFP (pEGFP-C1). Two to three days after transfection, GFP-positive clones were detected and clonally expanded in 96-well plates. Two clones, 70.8 and 71.3, were selected for further experiments. Genomic DNA preparation was performed to amplify the gene-edited loci by PCR and to verify the deletion in the FLNA reading frame (Figure S1A). Deletion was also confirmed by sequencing of the affected sequence area (Figure S1B,C). For method details, see Appendix A.1.

### 2.4. Isolation of DNA, RNA and Protein

Extraction of total protein and isolation of mRNA from TCam-2 cells was described previously [24]. DNA was extracted using the Wizard^®^ SV Genomic DNA Purification System (Promega, Mannheim, Germany) according to the manual.

### 2.5. Reverse Transcription (RT), Conventional and Quantitative Real-Time PCR

First strand cDNA synthesis was carried out using dN12 random primers in a volume of 40 µL as described in [25]. Conventional PCR as well as quantitative real-time PCR (qPCR) were performed as described previously [25,26]. PPIA or L19 gene expression served as the internal control. Primer sequences are listed in Table S1.

### 2.6. Western Blotting

Immunoblotting of TCam-2 whole-cell lysates was performed as described [21,26]. Protein samples (15 µg) were separated by 10% SDS-PAGE and transferred to a nitrocellulose membrane (pore size 0.2 μm). Primary antibodies (see Table S2) were incubated overnight at 4 °C. β-actin (ACTB) served as the loading control. Proteins were visualized with secondary IRDye^®^ 680RD/800CW donkey anti-rabbit or anti-mouse secondary antibodies (1:10,000; LI-COR Biosciences, Lincoln, NE, USA) in blocking buffer for 1 h at room temperature (RT), and detected by Odyssey^®^ CLx (LI-COR Biosciences, Lincoln, NE, USA). Densiometric quantification of the Western blots was performed with Image Studio Lite™ (version 5.2.5, LI-COR Biosciences). For quantification, the target protein signal was normalized to the loading control β-actin (ACTB).

### 2.7. Immunohistochemistry

Isoform-specific polyclonal antibodies for human FLNA and FLNB were generated in a previous study [27]. FLNA-specific antibodies were raised against hinge region 2 plus repeat 24 of FLNA, and FLNB-specific antibodies against hinge region 1 of FLNB. For primary antibody details, see Table S2.

Immunohistochemical staining of slices from paraffin-embedded testicular samples of patients with normal spermatogenesis was performed according to the avidin–biotin–peroxidase method as described in [25]. Negative controls consisted of rabbit IgG (2 µg/mL; Millipore, Billerica, MA, USA) instead of the primary antibody, or of omission of the first antibody. After immunohistochemical staining, sections were counterstained with hematoxylin, and analyzed using a Zeiss Axioplan microscope (Carl Zeiss Microscopy, Jena, Germany), and digitalized (Jenoptik, Jena, Germany).

### 2.8. Live-Cell Imaging

TCam-2 WT and FLNA-deficient cells were grown overnight in a glass-bottomed culture dish (µ-Dish, Ø 35 mm; ibidi, Gräfelfing, Germany) under standard conditions (5% CO_2_, 37 °C, 95% relative humidity, 2% O_2_) using a heating and incubation system (ibidi) and recorded for 24 h (Axiovert 135; Carl Zeiss Microscopy). Images (ProgRes MF, Jenoptik, Jena, Germany) were taken every 20 min to create a time-lapse series (Micro-Manager 1.3 Microscopy Software Ron Vale’s laboratory at UCSF, San Francisco, CA, USA). Movie sequences were produced with iMovie 9.0.3 (Apple Inc., Cupertino, CA, USA).

### 2.9. Wound-Healing Scratch Assay and Shear Stress Experiments

A sample of 3 × 10^5^ per ml of WT or FLNA-deficient TCam-2 cells were seeded in an ibiTreat µ-Dish (ibidi) and grown for 24 h. The confluent cell monolayers were scraped with a 10 µL pipette tip to create a cell-free scratch, and the plates were washed once with 1 mL D-PBS to remove cell debris before covered with 1 mL of fresh RPMI medium (with 1% P/S and 10% FCS). Cell migration was recorded using an inverted microscope using a 5× objective (Axiovert 135; Carl Zeiss Microscopy), and images of the same field were captured at the indicated time intervals and the percentage of confluence (C) was quantified using the PHANTAST plug-in for Fiji [28] at 0 and 72 h. Each assay was repeated two times.

For shear-stress experiments (*n* = 2), 5 × 10^5^ WT or FLNA-deficient TCam-2 cells were plated overnight in a µ-Slide I^0.4^ Luer (ibiTreat, ibidi, Gräfelfing, Germany). Channel slides were placed in a stage incubation system set to 37 °C, 5% CO_2_ and 95% humidity (ibidi Stage Top Incubation System, Universal Fit) integrated into an inverted microscope (Zeiss, Axiovert 135 TV, camera (ProgRes^®^ MF cool, JENOPTIK, Jena, Germany). Cells were exposed to laminar flow (ibidi Pump System) at a shear force ranging from 5–35 dyn per cm^2^ for 90 min each (for details see Table S7). Time-lapse phase-contrast images (10× magnification) were taken every 5 min (ProgRes^®^ CapturePro 2.9.0.1), and confluence was measured using the PHANTAST plug-in for Fiji [28].

### 2.10. Immunofluorescence Microscopy

For immunolabeling, TCam-2 WT and FLNA-deficient cells settled onto round 12-mm glass coverslips were fixed with 15% picric acid/2% paraformaldehyde in 10 mM PIPES, pH 6.0, for 20 min and post-fixed with 70% ethanol for 10 min. Cells were then washed three times in PBS, once with 10 mM PIPES, and twice with PBS/1% glycine, and incubated in blocking buffer (PBS plus 2% bovine serum albumin) for 1 h at RT. After blocking, the cells were washed three times with PBS and incubated with primary antibodies (see Table S2) for 2 h at RT, followed by the incubation with secondary antibodies for 1 h at RT. DNA was visualized by staining with DAPI. After immunostaining, samples were washed three times in PBS and embedded using Dako mounting medium (Agilent Technologies, Santa Clara, CA, USA). For visualization of filamentous actin, cells were stained with Atto 568-phalloidin (Sigma Aldrich, St. Louis, MO, USA). Ladinin 1 was detected using a polyclonal anti-LAD1 antibody (Table S2) followed by labeling with Atto 488-conjugated goat anti-rabbit IgG (Thermo Fisher Scientific, Waltham, MA, USA). See Table S2 for antibody details.

Images were acquired using an Observer Z1 microscope (Carl Zeiss Microscopy) equipped with a 63× PlanApo oil immersion objective (1.40 NA) and ZEN acquisition software. Several hundred cells were analyzed for each immunofluorescence staining, and representative images are shown.

### 2.11. Immunoprecipitation

Immunoprecipitation (IP) was performed using Protein G Sepharose beads (Sigma-Aldrich, Taufkirchen, Germany) according to the manual. Total protein lysate was prepared from 5 × 10^6^ TCam-2 cells using RIPA buffer (Cell Signaling Technology, Frankfurt am Main, Germany) supplemented with protease inhibitor mix (Roche cOmplete, Sigma-Aldrich). For each IP reaction, 125 µL Protein G beads and 10–20 µL serum were used. Antibody binding was performed on ice for 30 min. Immunoprecipitation of target antigens was performed at 4 °C for 90 min. For LC-MS/MS mass spectrometry, beads were washed and digested on the bead using trypsin [29]. LC-MS/MS mass spectrometry was conducted by the Protein Analysis Core Facility of the Biomedical Center (LMU).

For Western blot analysis, Protein G beads were separated from the supernatant by centrifugation, washed and bead–protein complexes were resuspended in 120 μL SDS loading buffer.

### 2.12. Affymetrix/Illumina HT-12v4 Expression Arrays

The Affymetrix expression array analysis of germ cell tumor (GCT) tissues (seminomas, *n* = 4; embryonic carcinoma (ECs), *n* = 3; teratomas, *n* = 3; germ cell neoplasia in situ (GCNIS), *n* = 4; mixed GCTs, *n* = 4; normal testis tissue (NTT), *n* = 4) was performed previously and re-analyzed in the context of this study [30]. Illumina microarray expression data of parental GCT cell lines (TCam-2, *n* = 5; 2102EP, *n* = 5, NCCIT, *n* = 4; JAR, *n* = 2; FS1, *n* = 4) were extracted from previous studies available via GEO (ncbi.nLm.nih.gov/geo/) (GSE71239, GSE71269, GSE79065, GSE60698) [31,32,33,34,35].

### 2.13. Mass Spectrometric Analysis

Lysis of cell pellets was done in 8 M Urea/400 mM NH_4_HCO_3_ by 2 min of sonication (Sonopuls GM3200 with BR30 cup booster, Bandelin, Berlin, Germany). For each sample, 25 µg of protein were reduced for 30 min at 37 °C with dithioerythritol (final concentration 5 mM), and alkylated using iodoacetamide (30 min, final concentration 15 mM). Proteins were digested for 4 h at 37 °C using Lys-C (FUJIFILM Wako Chemicals Europe, Germany) at an enzyme/substrate ratio of 1/100. After 8-fold dilution with water, proteins were further digested overnight using modified porcine trypsin (Promega, Madison, WI, USA) at an enzyme/substrate ratio of 1/50 at 37 °C. For proteome analysis, 1 µg of peptides was injected on an Ultimate 3000 nano-chromatography system (Thermo Scientific, Waltham, MA, USA) and separated at 250 nL/min using a 50 cm separation column (PepMap RSLC C18 2 µm 100 Å 75 µm × 50 cm). As solvent A, 0.1% formic acid in water and as solvent B, 0.1% formic acid in acetonitrile were used. The chromatography runs consisted of 160 min gradients from 3% to 25% solvent B, followed by 10 min gradients from 25% to 40% solvent B. MS spectra were collected using a top 15 data-dependent method on a Q Exactive HF X mass spectrometer. For protein identification, MaxQuant (v.1.6.7.0) [36], and the human subset of the UniProtKB/Swiss-Prot database (v.2020-03-30) were used. MS raw data from technical replicates were merged for both biological replicates. Identifications were filtered with a false discovery rate (FDR) < 0.01 at the peptide and protein level. Downstream data analysis was performed in Perseus (v.1.6.7.0) [37]. Peptide sequence coverage for FLNA and FLNB was visualized with Peptigram [38].

### 2.14. Statistics

A Student’s *t*-test was used to compare cell numbers of WT versus FLNA-KO TCam-2 cells. The criterium for statistical significance was *p* < 0.05 (*). For the determination of mRNA levels, samples were pipetted in duplicate, and qPCR runs were performed twice to generate mean values of two clones + SEM.

## 3. Results

### 3.1. FLNA and FLNB Detection in Human Testicular Biopsies of Adult Men with Normal Spermatogenesis

**Figure 1:**
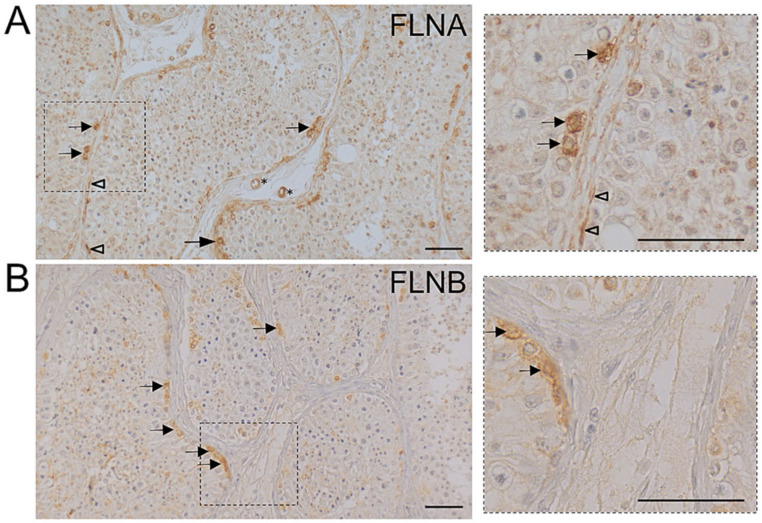
Immunohistochemistry of human testicular biopsies using isoform-specific antibodies directed against filamin A (FLNA) or filamin B (FLNB). (**A**) FLNA staining is found in peritubular, myoid cells (arrow heads in (A) and the enlargement of the boxed area on the right) building the wall of seminiferous tubules as well as in spermatogonia (arrows in (A) and the enlargement of the boxed area on the right). Some cells, presumably Leydig cells and/or macrophages scattered in the interstitial compartment, as well as endothelial cells of blood vessels (asterisks in (**A**)), are also slightly immunopositive. (**B**) FLNB immunostaining is restricted to spermatogonial cells (arrows in (**B**) and in the enlargement of the boxed area on the right) while peritubular, myoid cells are devoid of staining by FLNB-specific antibodies. Nuclei are slightly stained with hematoxylin. Scale bars: 50 µm.

Immunohistochemical analysis of human testicular biopsies using specific antibodies directed against FLNA or FLNB revealed that the two filamin isoforms have partly overlapping regions of localization. In detail, FLNA is expressed by peritubular myoid cells forming a wall around the seminiferous tubules, and is strongly enriched in both individual spermatogonia and also clustered spermatogonia (Figure 1A). Some slightly stained cells, presumably Leydig cells and/or macrophages scattered in the interstitial compartment, were also observed. In addition, cells of blood vessels were FLNA positive. FLNB immunostaining (Figure 1B) was restricted to spermatogonia (Figure 1B), while peritubular myoid cells were devoid of staining. In contrast, FLNC was not detected in human testicular slices (data not shown). The staining results were identical in all four testicular samples examined. No labeling was detectable in control sections when the primary antibody was substituted by IgG.

### 3.2. FLNA and FLNB Expression in Human Testicular Cancer Tissues and TCam-2 Cells

**Figure 2:**
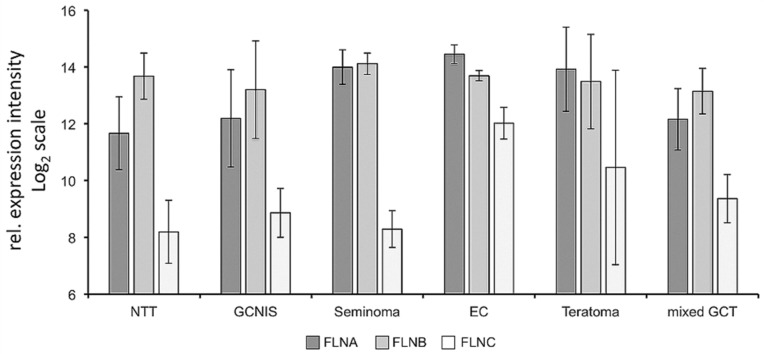
FLNA and FLNB are found with high prevalence to be associated with human testicular cancers. The histograms depict cDNA microarray expression data of FLNA, FLNB and filamin C (FLNC) genes in germ cell tumor (GCT) tissues (germ cell neoplasia in situ (GCNIS), seminoma, embryonic carcinoma (EC), teratoma and mixed GCT). Normal testis tissue (NTT) served as control. Means ± SEM of three to four individual samples.

We tested the expression of FLNA, FLNB and FLNC in different germ cell cancer tissues using an Affymetrix expression array analysis and found abundant expression of FLNA, in particular in seminonas, embryonic cancers and teratomas (Figure 2). Expression of all filamins was also detected in TCam-2 cells (data not shown).

Proteomic analyses of total cell lysates from seminoma-derived TCam-2 cells identified a total of 4408 proteins at FDR < 0.01 (Table S3). Using MaxQuant label-free quantification, FLNA and FLNB were among the top 50 most abundant proteins in the TCam-2 cell proteome, and were identified with 116 and 130 peptides (Tables S4 and S5), corresponding to a sequence coverage of 64.1% and 71.3%, respectively (Figure S2). These results show that both FLNA and FLNB are highly abundant proteins.

### 3.3. Identification of FLNA-Interacting Proteins in TCam-2 Cells

In order to identify FLNA-interacting proteins in TCam-2 cells, we performed an immunoprecipitation study using FLNA-specific antibodies (Figure S3). Mass spectrometric analyses identified a number of proteins that either directly or indirectly interact with FLNA (Table 1 and Table S6). We did not aim to identify the complete set of FLNA-interacting proteins, but were rather interested in cytoskeletal and regulatory proteins that might play specific roles in TCam-2 cells. Among these proteins were the cytoskeletal proteins FLNB, actin and cofilin-1, for which a direct interaction with FLNA was described previously (thebiogrid.org), as well as ezrin, zyxin and epiplakin (Table 1).

In addition, we identified ladinin 1 (LAD1), deleted in azoospermia-associated protein 1 (DAZAP1, DAZ-associated protein 1) and RuvB-like 1 (RUVBL1) as new interactors of FLNA. Interestingly, these proteins have been described previously as playing a role for either tumorigenicity or spermatogenesis, but based on the approach used in the present work, it is not possible to detail the nature of the interaction with FLNA. However, LAD1 was described only recently as a new FLNA-binding regulator of actin dynamics in response to epidermal growth factor (EGF) in MCF10A-immortalized mammary cells [39]. This study revealed a physical interaction of LAD1 with FLNA, and showed that LAD1 affects signaling and transcriptional networks. Depletion of LAD1 is associated with slower rates of cell proliferation and a reduction of tumorigenicity [39].

Deleted in azoospermia-associated protein 1 (DAZAP1) is an RNA-binding protein required for development and spermatogenesis in mice [40]. It has been implicated in RNA transcription, splicing and translation. It is highly expressed in testes, predominantly in late stage spermatocytes and post-meiotic spermatids. DAZAP1 deficiency in mice results in growth retardation and spermatogenic arrest. DAZAP1 interacts with germ cell-enriched deleted in azoospermia-like (DAZL), recently shown to be important for spermatogenesis and germ cell development [41]. In general, DAZ family proteins have been described as key players in germ cell development [42].

RUVBL1 (INO80H, TIP49a) is a triple A DNA helicase and one component of the chromatin-remodeling INO80 complex, which is involved in transcriptional regulation. RUVBL1 can form oligomers together with RUVBL2 (INO80J, TIP48) that we also detected in one immunoprecipitate (Table S6). For mice and rats, RUVBL1 has been shown to be highly expressed in testes. In addition, RUVBL1 is abundant in seminiferous tubules, where its location is restricted to germ cells [43].

### 3.4. Generation and Characterization of TCam-2 Cells Deficient in FLNA

**Figure 3:**
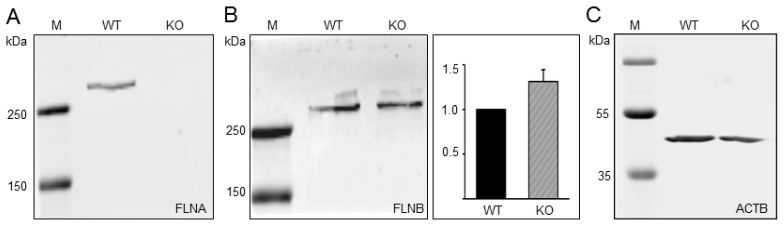
Western blot analysis of FLNA-deficient TCam-2 cells generated by CRISPR/Cas9 technology. (**A**) Cell lysates prepared from TCam-2 wildtype (WT) and FLNA-deficient (KO) cells were separated by SDS-PAGE, blotted and probed for the presence of (**A**) FLNA or (**B**) FLNB with isoform-specific antibodies. (**C**) After stripping, the blots were probed with anti–β-actin (ACTB) antibodies (only shown for FLNA). The experiment was repeated three times with very similar results. The quantification of FLNB protein levels was normalized to the levels of the loading control ACTB.

To assess the role of FLNA in TCam-2 seminoma cells, FLNA was deleted using CRISPR/Cas9-mediated gene editing. FLNA-deficient clones were identified by PCR analysis and DNA sequencing as well as Western blot analysis (Figure 3, Figure S1). PCR analysis of the clone 70.8 revealed deletion of both alleles of the FLNA gene and showed no band corresponding to the wildtype FLNA sequence (Figure S1A). Sequence analysis of both the WT and knockout (KO) amplicons further validated the gene editing in clone 70.8, as depicted by the electropherogram (Figure S1B) and the edited sequence (Figure S1C). The efficacy of the FLNA-KO was further confirmed by Western blot analysis (Figure 3). In detail, FLNA protein was detectable in WT, while it was undetectable in the FLNA-KO cells (Figure 3A). In contrast, the FLNB protein level showed a slight increase in FLNA-KO compared to TCam-2 WT cells (Figure 3B), suggesting that FLNB is not considerably upregulated to compensate for the deficiency of FLNA.

### 3.5. Deletion of FLNA Accelerates Cell Proliferation Rates, But Impairs Migration, Adhesion and Cell Morphology of TCam-2 Cells

**Figure 4:**
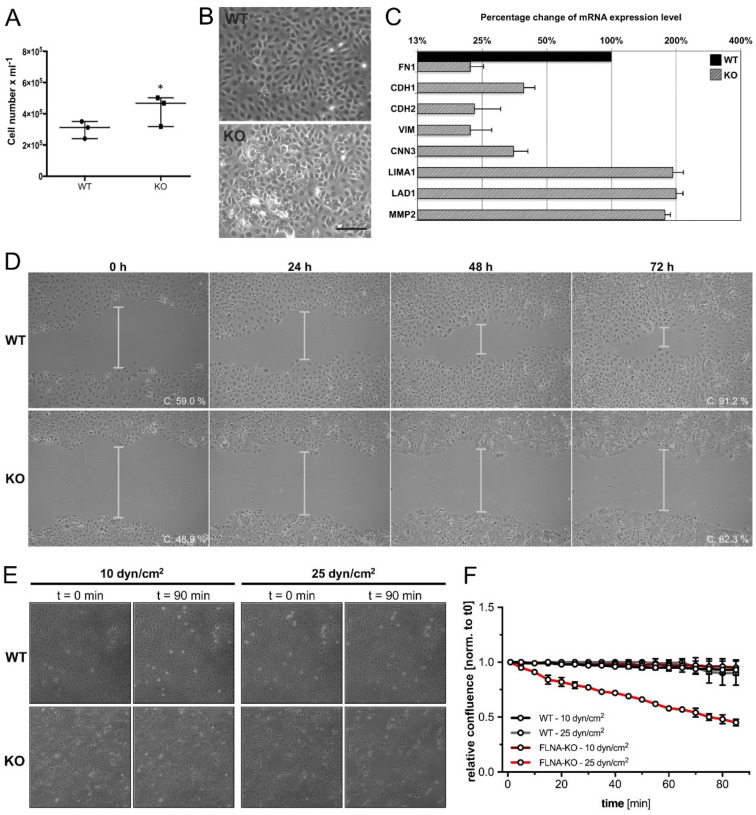
Characterization of FLNA-deficient TCam-2 cells. (**A**) FLNA-deficient TCam-2 cells (KO) show significantly (* *p* < 0.05) accelerated cell proliferation, compared to TCam-2 wildtype cells (WT), indicated by higher cell counts after 24 h. (**B**) In comparison to TCam-2 WT cells that show a primarily two-dimensional growth behavior, FLNA-KO cells are characterized by the tendency to grow three-dimensionally before confluence when plated on culture dishes for 24 h. Scale bar: 200 µm. (**C**) Deletion of FLNA is associated with altered mRNA levels of genes encoding cytoskeletal proteins, cell adhesion proteins and proteins involved in cell migration. Data are represented as percentage change of mRNA expression level of TCam-2 WT cells (black bar = 100%) compared to FLNA-KO cells (gray bars). Samples were pipetted in duplicate, and qPCR runs were performed twice to generate mean values of two clones + SEM. (**D**) Representative phase contrast images of a scratch assay performed with TCam-2 WT and FLNA-KO cells after 0, 24, 48 and 72 h post scratching. Percentage of confluence (C) at 0 and 72 h is indicated. Supplementary Videos S1 and S2 show the corresponding time lapse recordings for TCam-2 WT and FLNA-KO cells. (**E**) Shear stress experiment with TCam-2 WT and FLNA-KO cells. Representative phase contrast images depict detachment behavior of cells at two different shear rates (10 and 25 dyn per cm^2^) at two different time points (0 and 90 min). Supplementary Videos S3–S6 show the corresponding time-lapse recordings. (**F**) Relative confluence of WT and KO cells after 90 min shear-force exposure (10 and 25 dyn per cm^2^). Experiments were performed twice.

In TCam-2 cells, deletion of FLNA significantly (*p* < 0.05) accelerated cell proliferation in comparison to WT cells (Figure 4A). In phase contrast microscopy studies, FLNA-deficient TCam-2 cells exhibited a markedly disturbed cellular morphology compared to WT cells when plated on culture dishes for 24 h (Figure 4B).

These data support the notion that in TCam-2 cells, FLNA plays a critical role in proper cell morphology, as well as in cell adhesion and motility. In light of that, we investigated in a first step the mRNA expression for a subset of genes related to cytoskeleton formation and function. Loss of FLNA strongly attenuated transcription rates of genes encoding vimentin (VIM) and calponin-3 (CNN3) compared to TCam-2 WT cells (Figure 4C). In a second step, and because cytoskeleton formation is intimately associated with cell adhesion and migration, these properties were analyzed in more detail by qPCR. Deletion of FLNA repressed mRNA levels of representative genes associated with cell adhesion and migration, such as fibronectin (FN1), cadherin 1 (CDH1) and cadherin 2 (CDH2) (Figure 4C). Yet, in contrast to these findings, transcripts for LIM domain and actin-binding protein 1 (LIMA1), LAD1 and the extracellular ubiquitous matrix metalloproteinase MMP2 were overexpressed in FLNA-deficient cells (Figure 4C).

To further investigate the involvement of FLNA in cell migration, a cell scratch migration assay was performed. As shown in Figure 4D, FLNA-deficient cells are much less efficient at migrating directionally and show decreased capacity to close the wound scratch compared to TCam-2 WT cells within the observation period of 72 h. Quantification of confluence (C, expressed as percentage of confluence) after 72 h compared to 0 h via the PHANTAST plug-in confirmed that FLNA-KO cells are much less able to close the gap as compared to WT cells (0 h: 59.0% WT compared to 48.9% KO; 72 h: 91.2% WT compared to 62.3% KO). Supplementary Videos S1 and S2 show the corresponding time-lapse recordings for TCam-2 WT and FLNA-deficient cells.

Moreover, laminar flow experiments revealed a difference in response to shear force between TCam-2 WT and FLNA-KO cells. While most TCam-2 WT cells remained attached to the surface at 25 dyn per cm^2^ (after 90 min), many of the FLNA-KO cells were already detached (Figure 4E). Quantitative evaluation of adhesiveness (expressed as relative confluence) of TCam-2 WT and FLNA-KO cells after 90 min of shear-force exposure corroborates this finding (Figure 4F).

### 3.6. Immunofluorescence Analysis Shows Impaired Arrangement of Cytoskeletal Structures in FLNA-Deficient Cells in Comparison to TCam-2 Wildtype Cells

**Figure 5:**
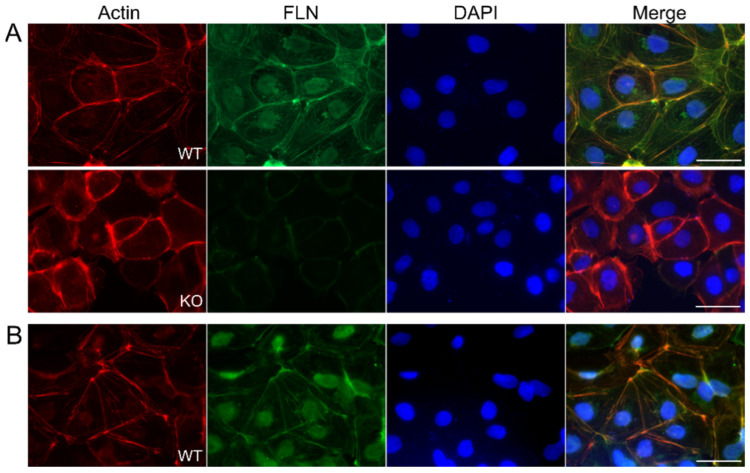
Fluorescence microscopy of TCam-2 wildtype (WT) and FLNA-deficient (KO) cells. (**A**) TCam-2 WT (WT) and FLNA-KO cells (KO) were stained with Atto 568-phalloidin for actin (red), immunolabeled for FLNA (green) using FLNA-specific and Alexa-488-labeled secondary antibodies and DAPI to visualize nuclei (blue). Merged images are shown on the right. (**B**) Immunofluorescence localization of FLNB (green) in TCam-2 WT cells. Scale bars: 50 µm.

FLNA, as an actin-crosslinking protein, has been implicated in the maintenance of the three-dimensional cortical actin network, and thus in cellular integrity and withstanding forces. Immunocytochemistry of TCam-2 WT cells showed that both FLNA and FLNB localize along filaments and decorate cell edges, and thus largely overlap with the regular distribution of filamentous actin (Figure 5A,B). Immunocytochemistry of FLNA-deficient TCam-2 cells revealed that in the absence of FLNA, structural elements containing filamentous actin are more irregularly arranged and disorganized overall (Figure 5A, lower panel). Furthermore, FLNA-deficient cells are characterized by more loose and interrupted contact areas with neighboring cells. In contrast, TCam-2 WT cells form straight contacts delineated by underlying actin bundles. Hence, loss of FLNA is obviously sufficient to impede the well-organized distribution of filamentous actin.

**Figure 6:**
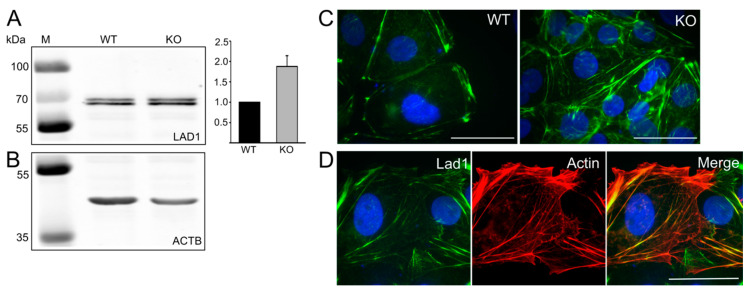
Expression and localization of LAD1 in TCam-2 wildtype (WT) and FLNA-deficient (KO) cells. (**A**) Western blot analysis of LAD1 in TCam-2 WT and FLNA-KO cells. (**B**) Loading control ACTB. LAD1 protein levels were normalized to the loading control ACTB and the quantification revealed an increase in LAD1 (histogram on the right). The upper band detected by the LAD1 antibody most probably represents phosphorylated LAD1 [39]. (**C**) Immunofluorescence localization of LAD1 in TCam-2 WT and FLNA-KO cells. (**D**) Partial co-localization of filamentous actin (red) and LAD1 (green) in FLNA-KO cells. Scale bars: 50 µm.

Immunoprecipitation studies indicated that LAD1 is one potential FLNA interaction partner (Table 1). The mRNA expression level of LAD1 in FLNA-KO cells indicated an approximately twofold increase. Western blot analysis of the LAD1 protein level in WT and FLNA-KO TCam-2 cells confirmed the increased LAD1 protein level (Figure 6A,B). Immunofluorescence microscopy showed that in TCam-2 WT cells, LAD1 localizes along the cell boundaries where filamentous actin is detected and is enriched preferentially at the ends of stress fibers (Figure 6C). In FLNA-KO cells, LAD1 still colocalizes with filamentous actin structures, but is more irregularly distributed (Figure 6C,D). In particular, clearly defined, straight, belt-like cell boundaries are not detected any more, and, together with loosened substrate contact, may result in a more three-dimensional cell growth behavior.

### 3.7. Deletion of FLNA Causes Altered Stem Cell Characteristics

**Figure 7:**
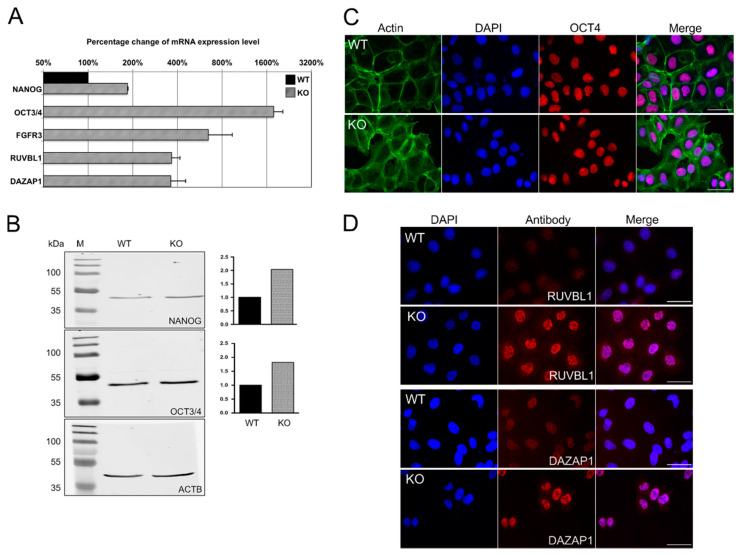
Increased expression of stem cell markers in TCam-2 cells deficient in FLNA (KO) compared to wildtype (WT) cells. (**A**) Deletion of FLNA causes increased levels of mRNAs encoding the embryonic stem cell markers NANOG, OCT3/4 and FGFR3, as well as RUVBL1 and DAZAP1. mRNA data are depicted as percentage change of mRNA expression levels of TCam-2 WT cells (WT, black bar = 100%) compared to FLNA-deficient cells (KO, gray bars). Samples were pipetted in duplicate and qPCR runs were performed twice to generate mean values of two clones; + SEM. (**B**) Western blot analysis of NANOG and OCT3/4. The quantification of protein normalized to levels of ACTB confirmed increased protein levels for both markers in FLNA-KO cells. Quantification of the Western blot shown is presented on the right. (**C**) Immunofluorescence microscopy of OCT4 in TCam-2 WT and FLNA-KO cells. (**D**) Immunofluorescence localization of RUVBL1 and DAZAP1 in TCam-2 WT and FLNA-KO cells. Scale bars: 50 µm.

qPCR measurements revealed that FLNA-deficient cells show enhanced transcript levels of the stem cell markers OCT3/4, NANOG and FGFR3 in comparison to TCam-2 WT seminoma cells (Figure 7A). The increased expression of NANOG and OCT3/4 in KO cells was supported by Western blot analysis (Figure 7B), and presence of OCT4 was confirmed by immunofluorescence (Figure 7C).

Similar to these results, transcripts of the FLNA interactors RUVBL1 and DAZAP1, supposed to be associated with spermatogenesis, were also elevated in FLNA-deficient cells in comparison to TCam-2 WT cells (Figure 7A). This finding was further supported by immunofluorescence analysis. While RUVBL1 and DAZAP1, in contrast to OCT4 (Figure 7C), were barely detectable in interphase TCam-2 WT cells, antibodies directed against RUVBL1 and DAZAP1 labeled nuclei of FLNA-deficient cells (Figure 7D).

## 4. Discussion

Our current understanding of FLNA functions originates from the investigation of different primary and established cell lines, mouse knockout models and *FLNA* mutations in humans. Mouse knockout models have shown that FLNA is critical for skeletal, vascular, muscular, cardiac and cerebral development [8], and a number of clinical case reports described and partially analyzed the molecular pathology of the different facets of the diseased state [11,44]. Male knockout mice die before birth with vascular defects [45]. This is one of the reasons why our knowledge about functions of FLNA in the testis is very limited.

One of the few studies that contributed to the understanding of FLNA’s role in the vertebrate testis was carried out in rats. The authors provided evidence that FLNA is involved in the assembly of the blood–testis barrier during the postnatal development in rat testis [46]. A recent study [47] described FLNA in testes of mice in germ cells and peritubular cells. The data are in general agreement with respect to peritubular cells. Our previous proteomic studies of human testicular peritubular cells had readily identified FLNA [23,48]. These cells are structural, myofibroblastic cells and the expression of FLNA is therefore not unexpected. The present study, employing immunohistochemistry, confirms the expression of FLNA in situ.

The results of the present study and the mentioned study in mice [47] differ, however, with respect to germ cell expression of FLNA. We found expression mainly in spermatogonia, while in mice it was detected in elongating spermatids. Species differences, especially in the testis, are not entirely unexpected, and a recent gene expression study revealed that among the organs examined, testis and liver showed the largest differences of individual genes between mice and humans compared to all other organs [49].

The human testis, and spermatogonia in particular, are not readily accessible for experimental studies. Because expression was also detected in human germ cells tumors, namely seminoma samples, we concentrated on the seminoma cell line TCam-2, which is related to spermatogonia [21]. Proteomic analyses of TCam-2 cells revealed that FLNA, and also FLNB, are highly abundant proteins.

FLNA has been shown to support oncogenic phenotypes in different tissues, summarized in a recent review [50]. Due to FLNA’s involvement in cytoskeletal reorganization, cell shape modeling, cell matrix interaction and migration, it is obvious that impairment of FLNA functionality may be relevant for cancer development and progression to metastasis. However, during recent years, it became clear that the role of FLNA in cancer is more complex [51], and a number of studies have shown that high levels of FLNA can have a tumor-promoting effect, or, on the contrary, can suppress tumor growth. It is believed that signaling factors that interact with FLNA, as well as the subcellular localization, determine the role of FLNA in cancer [51,52].

FLNA is known to mediate the interaction between cytoskeletal proteins that control cell adhesion [53]. One of the best studied interactions is the binding of FLNA to the cytoplasmic tail of the integrin β-chain. It was shown that an increased binding of FLNA to integrin results in a diminished migration [54]. During tumor progression, mechanical and structural changes occur not only intracellularly, but also within the extracellular matrix. It is thought that these changes in the microenvironment may promote malignancy and metastasis.

In order to elucidate functions of FLNA in seminoma cells, we have generated FLNA-deficient TCam-2 cells. The phenotypic investigation of FLNA-deficient cells revealed higher proliferation rates and indicated a transition from a two-dimensional to a three-dimensional growth behavior. TCam-2 cells lacking FLNA are less adherent, show migration defects in scratch assays and detach more easily under shear stress conditions. Both the cytoskeletal architecture and the cellular integrity are disrupted. These changes are accompanied by transcriptional changes. Transcripts for adhesion-mediating proteins like cadherins are reduced, whereas transcripts for metalloproteinases required for interstitial migration are increased. Transcription of genes encoding compensating cytoskeletal proteins, such as LIMA1, is also increased.

Our immunoprecipitation experiments aimed at the identification of direct and indirect FLNA interactors in order to define the ‘FLNA interactome’ in TCam-2 cells. In addition to cytoskeletal proteins that were already described as interacting with FLNA, we identified LAD1 as one potential FLNA-binding protein in TCam-2 cells. Although this interaction was not confirmed by further biochemical studies, it is very likely that LAD1 interacts directly with FLNA. LAD1 was identified recently as an interactor of FLNA in mammary cells and may be a marker of aggressive breast tumors [39]. In line with that, TCam-2 cells lacking FLNA overexpress LAD1, and its localization in FLNA-deficient cells largely overlaps with filamentous actin structures, indicating a disturbed cellular morphology. Detection of RUVBL1 and DAZAP as FLNA interactors indicates that FLNA is involved in the modulation of transcriptional processes via these proteins.

Taken together, the results obtained in this study imply that FLNA is critically involved in the cellular phenotype and growth of the seminoma cell line TCam-2, and possibly also plays an important regulatory role in human seminomas. Our immunohistochemical studies also indicated expression of FLNA in spermatogonia in situ in the adult human testis. We typically found pairs or larger groups of FLNA-positive spermatogonia. Spermatogonial stem cells (SSCs) reside in a niche and give rise to daughter cells that migrate away [55]. Whether some of the spermatogonia positive for FLNA (or FLNB) are SSCs remains to be investigated. Yet, FLNA expression may be typical for (some of) these cells during the process of differentiation. Further, the increases in OCT3/4, NANOG and FGFR3 in TCam-2 cells lacking FLNA are noteworthy and may indicate involvement in determining “stemness”. Whether the results obtained in TCam-2 cells are transferable and can be applied to normal male germ cells remains to be shown. If so, FLNA would be a critical, and as yet over-looked regulator of spermatogenesis in humans.

In summary, our data provide evidence that FLNA is crucially involved in balancing stem cell characteristics and invasive properties in human seminoma cells, and possibly human spermatogonial cells and SSCs. 

## Figures and Tables

**Table 1:** FLNA-binding proteins identified by immunoprecipitation using FLNA-specific antibodies and mass spectrometric analysis.

Protein	Entry Name	Gene Name	Molecular Weight
Filamin B	FLNB_HUMAN	FLNB	278 kDa
Actin, cytoplasmic	ACTB_HUMAN	ACTB	42 kDa
Epiplakin	EPIPL_HUMAN	EPPK1	556 kDa
RuvB-like 1	RUVB1_HUMAN	RUVBL1	50 kDa
Ezrin	E7EQR4_HUMAN	EZR	69 kDa
Zyxin	ZYX_HUMAN	ZYX	61 kDa
Ladinin-1	LAD1_HUMAN	LAD1	57 kDa
DAZ-associated protein 1	DAZP1_HUMAN	DAZAP1	43 kDa
Cofilin-1	COF1_HUMAN	CFL1	19 kDa

The immunoprecipitation experiment was performed three times. The compiled mass spectrometry data set is shown in Table S6. Presence of RUVBL1, LAD1 and DAZAP1 in the immune precipitate but not the supernatant was confirmed in Western blot analysis (Figure S3) using specific antibodies (Table S2).

## Supplementary Materials

The following are available online at https://www.mdpi.com/2073-4409/9/12/2563/s1, Figure S1: Validation of FLNA CRISPR/Cas9 knockout clone 70.8 by PCR analysis. Figure S2: Sequence coverage of mass spectrometry-based FLNA and FLNB identification. Figure S3: Immunoprecipitation of proteins from TCam-2 total cell lysates using FLNA-specific antibodies. Table S1: Forward (For) and reverse (Rev) oligonucleotide primer sequences. Table S2: List of antibodies. Table S3: Proteins identified in human seminoma TCam-2 cells. Table S4: Identified FLNA (P21333) peptides in TCam-2 cells. Table S5: Identified FLNB (O75369) peptides in TCam-2 cells. Table S6: Result of mass spectrometry analysis of the FLNA immunoprecipitation (IP) experiments. Listed are all the proteins identified in three independent FLNA IPs (FLNA IP 1–3) and control. Table S7: Flow rate profile, pressure and various shear stress rates used for TCam-2 WT and FLNA-KO cells. Video S1: Time-lapse video of a scratch assay performed with TCam-2 WT cells (p47) corresponding to the images shown in Figure 4D. Video S2: Time-lapse video of a scratch assay performed with FLNA-KO cells (p52) corresponding to the images shown in Figure 4D. Videos S3–S6: Time-lapse videos of flow experiments shown in Figure 4E.

## Appendix A

### Appendix A.1. Detailed Methodology of CRISPR-Cas9 Mutant Generation

#### Appendix A.1.1. Guide RNA Design and Cloning

Analysis for putative CRISPR targets, including prediction of off-target sites, was performed using the CRISPR design tool (http://crispr.mit.edu/). Each DNA oligo duplex (see below) has 5′ overhangs (forward: cacc, reverse: aaac) designed to be directly cloned into the *Bbs*I-digested pX330 expression vector (pX330-U6-Chimeric_BB-CBh-hSpCas9 Addgene plasmid # 42230) as described below.

#### Appendix A.1.2. Digestion of pX330

pX330 was digested with *Bbs*I in a total volume of 20 µL containing 4 µg pX330 vector, 2 µL *Bbs*I enzyme, 2 µL Tango buffer for 1 h at 37 °C. The digested vector was recovered from a 0.8% low-melting temperature agarose gel and purified with a QIAprep Spin Miniprep Kit for subsequent ligation reaction.

#### Appendix A.1.3. Annealing of Complementary gRNAs Oligos and Ligation

Two sets of 20-mer double-stranded gRNA oligonucleotides (first set 5′-caccgaagcgggcagagttcactg-3′ and 5′-aaaccagtgaactctgcccgcttc-3′; second set 5′-caccgcttatgagtccccgtcacct-3′ and 5′-aaacaggtgacggggactcataagc-3′; Metabion, Munich, Germany) were produced by annealing each set in a thermocycler machine using the following program: 95 °C for 1 min, followed by slow cooling from 95 °C to 4 °C at a ramp rate of 0.03 °C per s. We selected two pairs of gRNA oligos to induce a deletion of approximately 260 bp in the FLNA gene. By use of appropriate primers flanking the 5′ and 3′ region of the gRNAs, this should allow for rapid detection and discrimination of KO clones from WT.

Each of the DNA oligonucleotide duplexes were ligated into *Bbs*I-digested px330 vectors in a volume of 10 µL containing 1 µg linearized pX330, 1 µL annealed oligonucleotides, 1 µL T4 ligase buffer (10×) and 1 µL T4 ligase (Thermo) at 16 °C overnight. Cloning of both gRNA sets was verified by DNA sequencing using U6 universal primer (Eurofins, Ebersberg, Germany).

#### Appendix A.1.4. Transformation in TOP10

For pX330 plasmid propagation, One Shot^®^ TOP10 chemically competent *Escherichia coli* were transformed by pipetting 5 μL of each ligation reaction directly into 50 µL competent cells according to the manufacturer’s instructions.

#### Appendix A.1.5. Transfection of TCam-2 Cells

Samples of 1 × 10^5^ TCam-2 cells per well were seeded onto a six-well-plate in media without antibiotics overnight. The next morning, cells were simultaneously transfected with the pX330 vector encoding for two different sets of gRNAs targeting the FLNA coding region using 1 µg plasmid DNA in combination with 5 µL FuGENE HD transfection reagent (ratio of 1:5). Up to 72 h after transfection, media were changed, and cells were plated into the wells of a 96-well plate at a density of 0.5 cell per well by limiting dilution. Two to four weeks later, some clonal expanded cells were grown to colonies, which were subsequently assembled for PCR analysis.

#### Appendix A.1.6. Genomic DNA Preparation and Verification of Gene Editing by PCR Analysis

A crude and rapid DNA preparation by lysis (50 mM KCl, 10 mM Tris, pH 8.3, 25 mM MgCl_2_, 0.45% NP40, 0.45% Tween 20, Proteinase K (20 µg/mL) of TCam-2 cells was performed and 2 µL of the supernatant were directly used in PCR to amplify gene-edited loci by PCR with the following primers flanking the 5′ and 3′ region of the gRNAs: forward 5′-ccaaactgaacccgaagaaa-3′ and reverse 5′-caccctgtgacttatccacgta-3′.
