# Supplementary material for: Filamin A Orchestrates Cytoskeletal Structure, Cell Migration and Stem Cell Characteristics in Human Seminoma TCam-2 Cells
Source: Cells. 2020 Nov 30;9(12):2563. doi: 10.3390/cells9122563 (PMC7761120; doi:10.3390/cells9122563)
Supplement: Supplementary file 1 [file cells-09-02563-s001.zip › clean_Rev_Welter_etal_ Supplementary figures+table+movie.docx]

**Supplementary Materials**


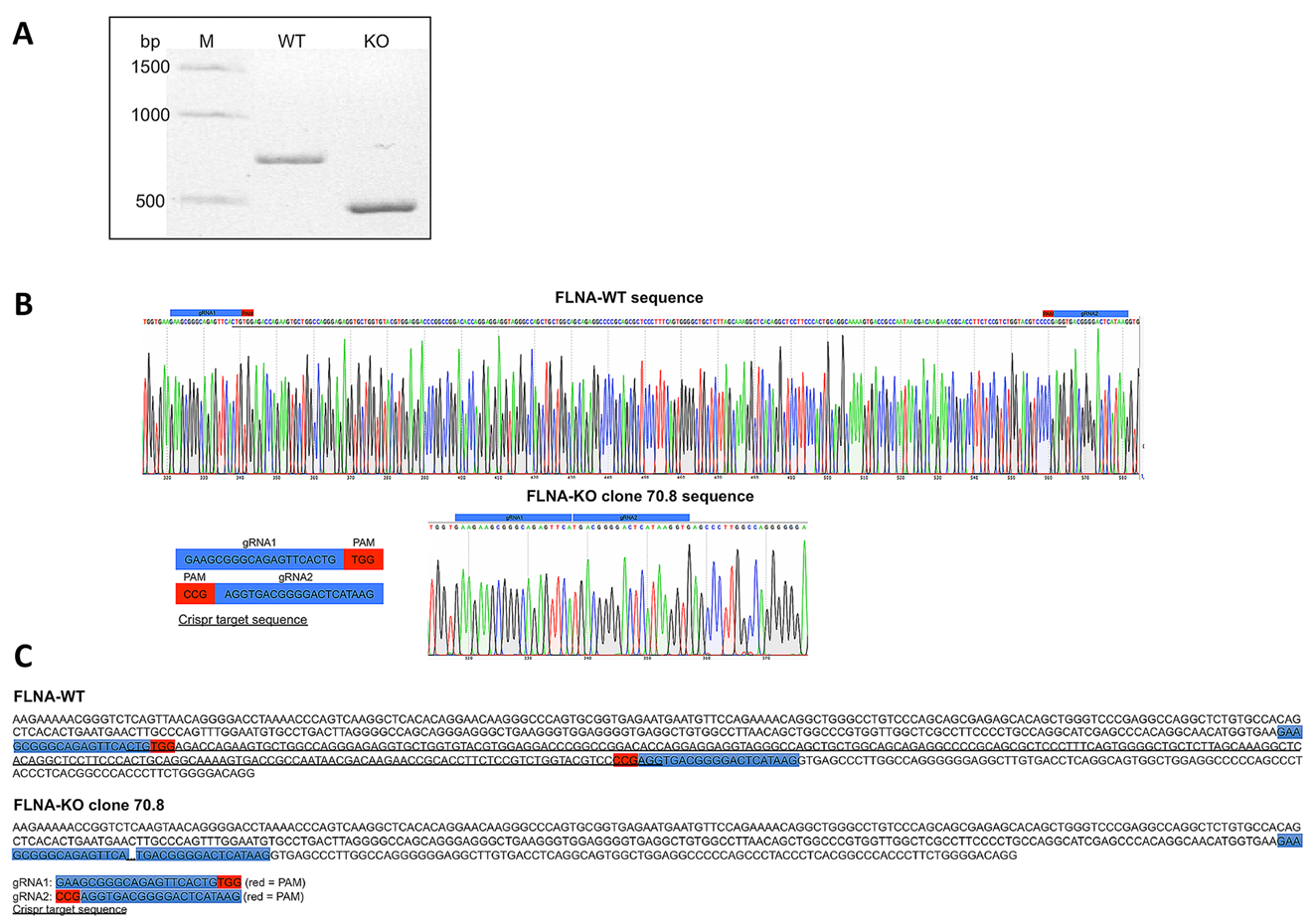


**Figure S1.** Validation of FLNA CRISPR/Cas9 knockout clone 70.8 by PCR analysis. (**A**) Shortened amplicon size of one subclone suggests the presence of mutations in the FLNA-gRNA target site. (**B**) Sequence analysis for TCam-2 WT and of the FLNA-targeted CRISPR KO-cells is depicted in the electropherogram, and (**C**) the final edited sequence. The PAM sequence (NGG), three bps upstream of the target, used to coordinate endonuclease activity is boxed in red; the gRNAs are boxed in blue, and the underlined sequence pinpoint the CRISPR-targeted FLNA-sequence.

**
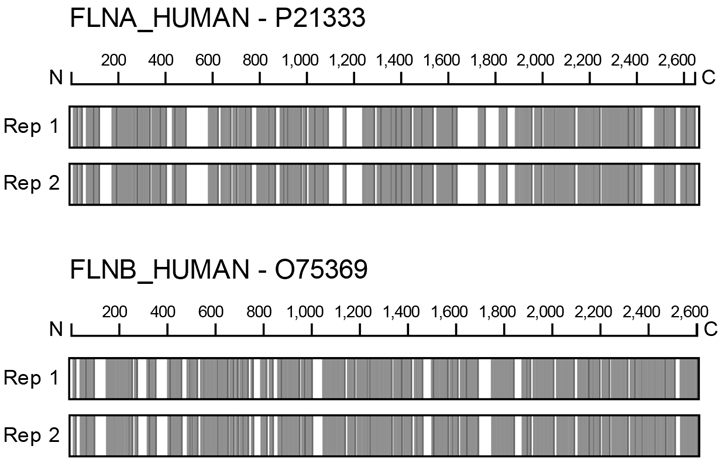
**

**Figure S2:** Sequence coverage of mass spectrometry-based FLNA and FLNB identification.

**
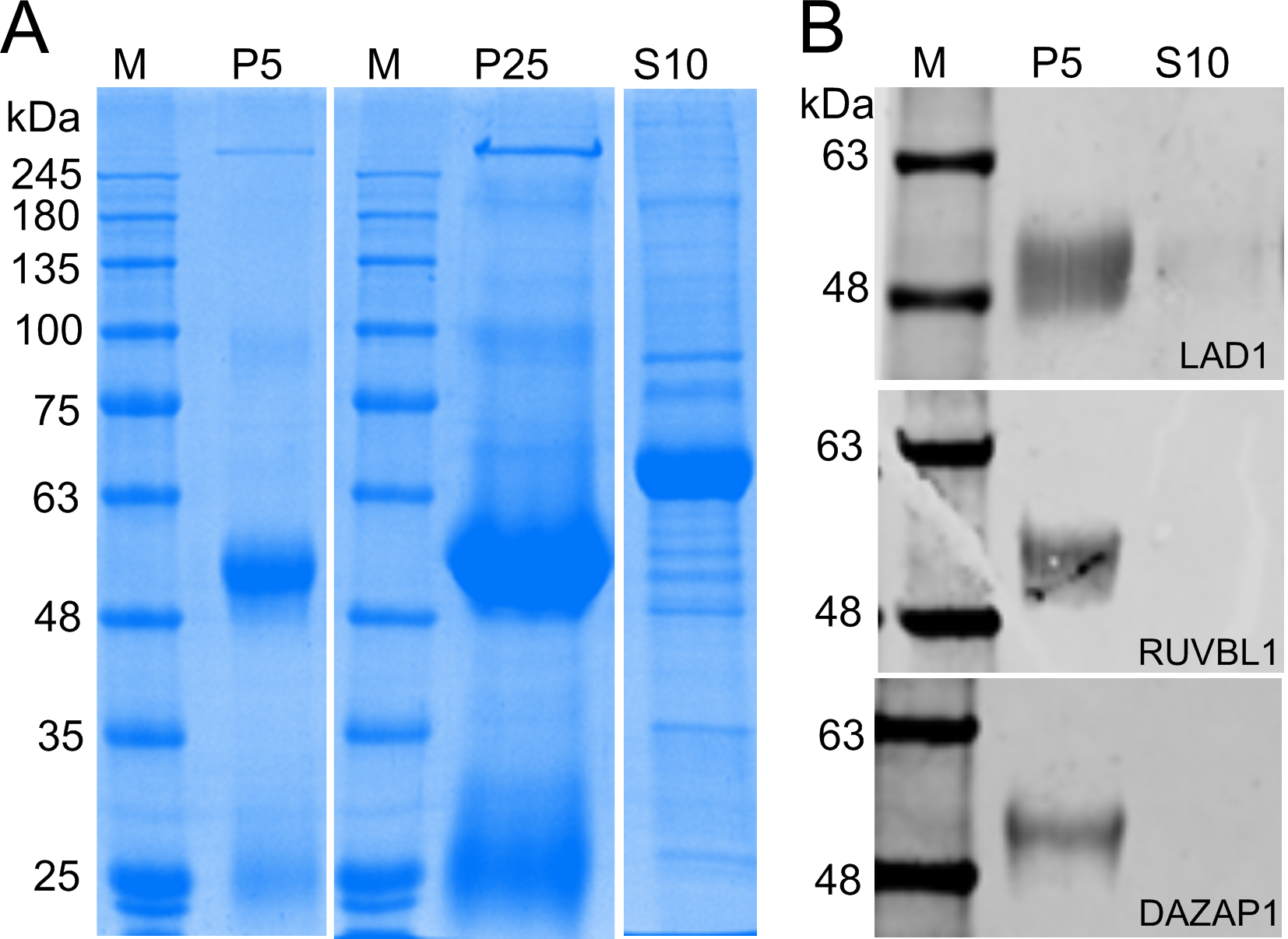
**

**Figure S3.** Immunoprecipitation of proteins from TCam-2 total cell lysates using FLNA-specific antibodies. (**A**) Immunoprecipitation (IP) was performed as described in Materials and Methods. 5 µl (P5) or 25 µl (P25) of the precipitate, or 10 µl of the supernatant (S10) were separated by 7 % SDS-gel electrophoresis and stained with Coomassie Brilliant blue R. (**B**) Western blot analysis of immunoprecipitated and supernatant proteins using the indicated antibodies.

**Table S1.** Forward (For) and reverse (Rev) oligonucleotide primer sequences (5´-3´) employed in real-time qPCR, and reference ID (accession number).

| **Gene Name** |  | **Sequence**  **(5´- 3´)** | **Reference ID**  **(accession number)** |
| --- | --- | --- | --- |
| L19 | For  Rev | AGGCACATGGGCATAGGTAA  CCATGAGAATCCGCTTGTTT | NM_000981.3 |
| PPIA | For  Rev | AGACAAGGTCCCAAAGAC  ACCACCCTGACACATAAA | NM_021130.5 |
| FN1 | For  Rev | TCCACAAGCGTCATGAAGAG  CTCTGAATCCTGGCATTGGT | AB191261.1 |
| CDH1 | For  Rev | GAACGCATTGCCACATACAC  ATTCGGGCTTGTTGTCATTC | NM_001317184.1 |
| CDH2 | For  Rev | TTTGAGGGCACATGCAGTAG  ACTGTCCCATTCCAAACCTG | NM_001792 |
| VIM | For  Rev | AGACAGGTGCAGTCCCTCAC  GCTTCAACGGCAAAGTTCTC | NM_003380.5 |
| CNN3 | For  Rev | AAGGATGGCATCATCCTCTG  GAGGCCAGTTCAGTGAGGAC | NM_001286056.1 |
| LIMA1 | For  Rev | GGAAATAGGCCCAGGTCAGT  TTTCTGAGAGGGTGGAAGT | NM_001113546 |
| LAD1 | For  Rev | AACCTACAGCTCCCTCA  TGCGAGTTAGGGTTGTTTCC | NM_005558.4 |
| MMP2 | For  Rev | TGATCTTGACCAGAATACCATCGA  GGCTTGCGAGGGAAGAAGTT | NM_004530.6 |
| FGFR3 | For  Rev | GTGCGGGTGACAGACGCTCC  CAGCGGAAGCGGACGGTGTT | NM_022965 |
| OCT3/4 | For  Rev | GAGAAGCTGGAGCAAAACCC  CGTTTGGCTGAATACCTTCCC | NM_001173531.1 |
| NANOG | For  Rev | TACCTCAGCCTCCAGCAGATGCAA  GCTGAGGCCTTCTGCGTCAC | NM_024865.2 |
| DAZAP1 | For  Rev | TCTTGACTGGAGCACGACC  ATGTTTCGGCCATCTAGCGT | NM_001352033.1 |
| RUVBL1 | For  Rev | GCATGTGGCGTCATAGTAGA  TCTCCTTTATTCGCAGCCCA | NM_001319084.1 |

**Table S2.** List of antibodies.

| Protein Name | Species | Dilution  WB | Dilution  ICC | Dilution  IHC | Source/ Product ID |
| --- | --- | --- | --- | --- | --- |
| FLNA | Rabbit | 1:5,000 | 1:800 | 1:500 | Roth et al., 2017 |
| FLNB | Rabbit | 1:1,000 | 1:800 | 1:500 | Roth et al., 2017 |
| FLNC | Rabbit | - | - | 1:100-500 | Sigma-Aldrich HPA006135 |
| OCT3/4 | Rabbit | 1:1,000 | 1:250 | - | Abcam #ab19857 |
| NANOG | Goat | 1:1,000 | 1:250 | - | Santa Cruz Biotechnology Sc-30331 |
| RUVBL1 | Mouse | - | 1:200 | - | Santa Cruz Biotechnology  Sc-393905 |
| DAZAP | Mouse | - | 1:200 | - | Santa Cruz Biotechnology  Sc-373987 |
| ACTB | Mouse | 1:5,000 | - | - | Sigma-Aldrich #32001A |
| LAD1 | Rabbit | 1:1,000 | 1:1,000 | - | Sigma-Aldrich HPA028732 |

**Table S7:** Flow rate profile, pressure and various shear stress rates applied in flow experiments with TCam-2 WT and FLNA-KO cells.

| **shear stress (dyn/cm^2^)** | **flow rate (ml/min)** | **pressure (mbar)** |
| --- | --- | --- |
| 5 | 5.44 | 10.3 |
| 10 | 10.85 | 21.2 |
| 15 | 16.29 | 32.9 |
| 20 | 21.69 | 45.3 |
| 25 | 27.14 | 58.7 |
| 30 | 32.55 | 73.0 |
| 35 | 38.00 | 88.5 |

**Tables S3-S6 and Videos 1-6 are available only online.**

**Table S3.** Proteins identified in human seminoma TCam-2 cells by mass spectrometry. Identification was performed on two biological replicates with three technical replicates each. Reverse hits, hits only identified per site, and potential contaminants were excluded. Identifications are sorted in descending order according to the MaxQuant LFQ intensity. Filamin A (FLNA) and Filamin B (FLNB) identifications are highlighted.

**Table S4.** Identified FLNA (P21333) peptides in TCam-2 cells. Start and end position indicate position of the peptide in the protein sequence. PEP: Posterior Error Probability of the identification. This value essentially operates as a p-value with smaller values being more significant. Score: Highest Andromeda score for the associated MS/MS spectra.

**Table S5.** Identified FLNB (O75369) peptides in TCam-2 cells. Start and end position indicate position of the peptide in the protein sequence. PEP: Posterior Error Probability of the identification. This value essentially operates as a p-value with smaller values being more significant. Score: Highest Andromeda score for the associated MS/MS spectra.

**Table S6.** Result of mass spectrometry analysis of the FLNA immunoprecipitation (IP) experiments. Listed are all the proteins identified in three independent FLNA IPs (FLNA IP 1-3) and control.

**Video 1****.** Time-lapse video of a scratch assay performed with TCam-2 wildtype cells (p47) corresponding the images shown Figure 4D. Recording was for 96 hours.

**Video 2.** Time-lapse video of a scratch assay performed with FLNA-deficient cells (p52; clone 70.8) corresponding the images shown Figure 4D. Recording was for 96 hours.

**Videos 3-6.** Time-lapse videos of flow experiments shown in Fig. 4E. Video 3 corresponds to TCam-2 WT cells at 10 dyn/cm^2^, Video 4 corresponds to TCam-2 FLNA-KO cells at 10 dyn/cm^2^, Video 5 to TCam-2 cells at 25 dyn/cm^2^, and Video 6 to TCam-2 FLNA-KO cells at 25 dyn/cm^2^. Images were taken every 5 min. Video rate is 5 frames per second.

| 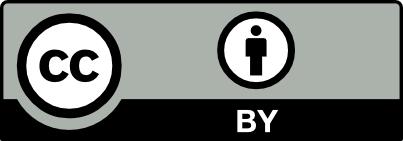 | © 2020 by the authors. Submitted for possible open access publication under the terms and conditions of the Creative Commons Attribution (CC BY) license (http://creativecommons.org/licenses/by/4.0/). |
| --- | --- |
