# Supplementary material for: Filamin A Orchestrates Cytoskeletal Structure, Cell Migration and Stem Cell Characteristics in Human Seminoma TCam-2 Cells
Source: Cells. 2020 Nov 30;9(12):2563. doi: 10.3390/cells9122563 (PMC7761120; doi:10.3390/cells9122563)

Original PCR-Data: Fig. S1

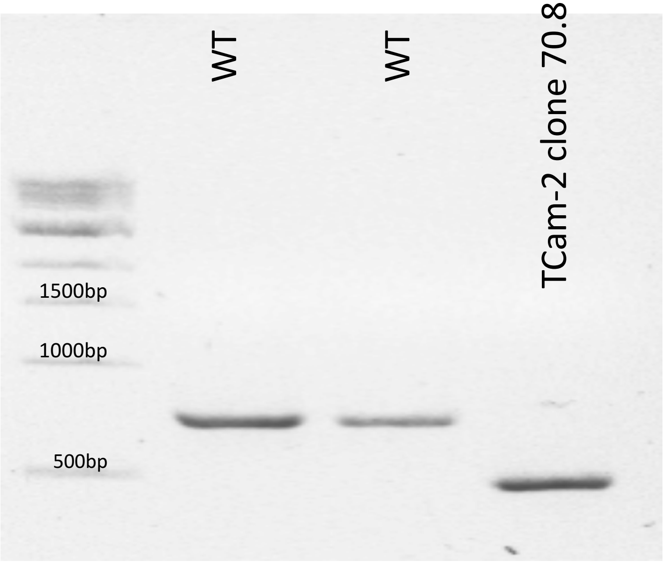

Original WB-Data: Fig. 3

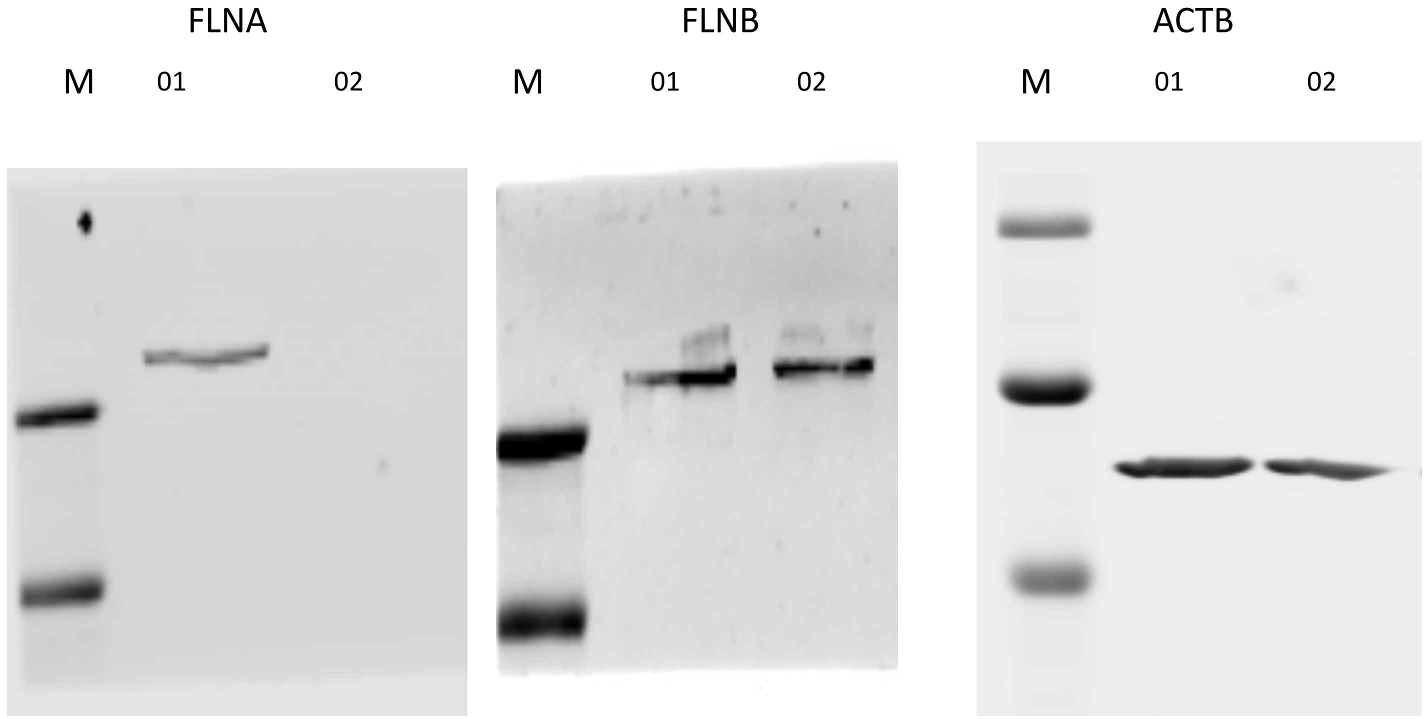

M: Marker
01: TCam-2 WT
02: TCam-2 FLNA-KO

Former Original WB-Data: Fig. 6

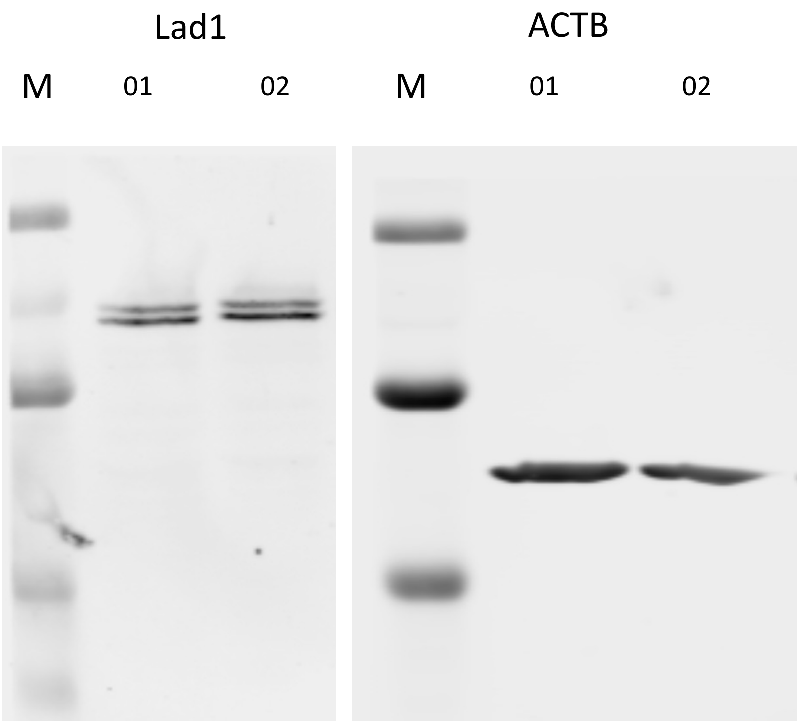

M: Marker
01: TCam-2 WT
02: TCam-2 FLNA-KO

New Original WB-Data: Fig. 6

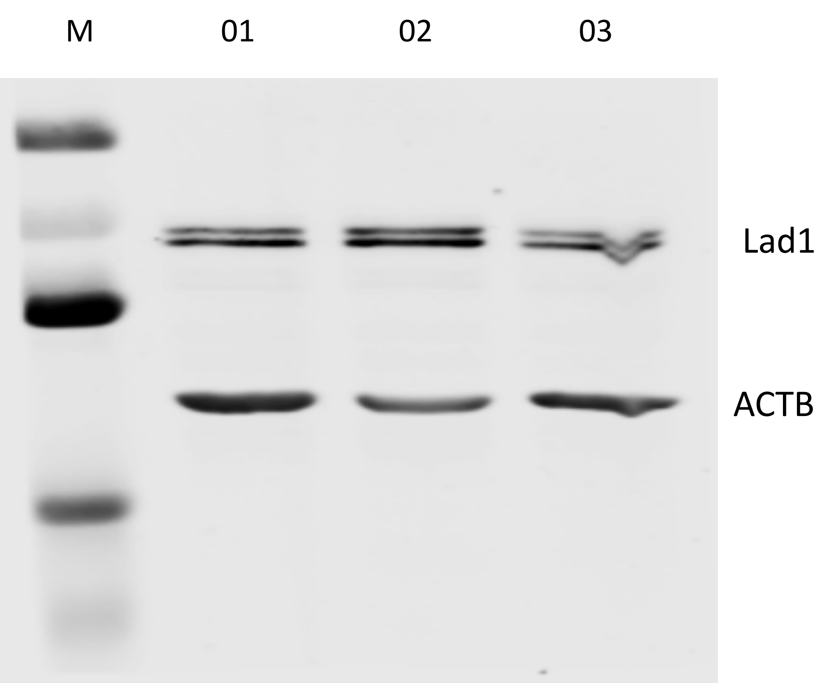

M: Marker
01: TCam-2 WT
02: TCam-2 FLNA-KO Clone 70.8
03: Tcam-2 FLNA-KO Clone 71.3

Original WB-Data: Fig. 7

01: TCam-2 WT 02: TCam-2 FLNA-KO

WB: Nanog

01 02

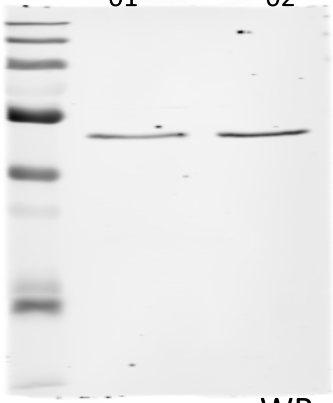

WB1: Oct3/4

01 02

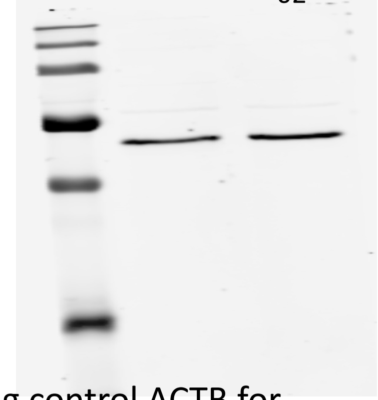

WB2: Oct3/4

01 02

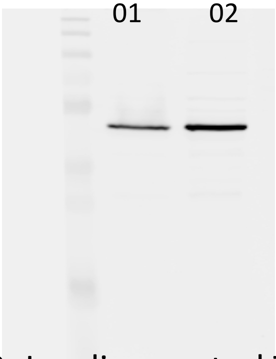

WB: Loading control ACTB for NANOG, WB1: OCT3/4

01 02

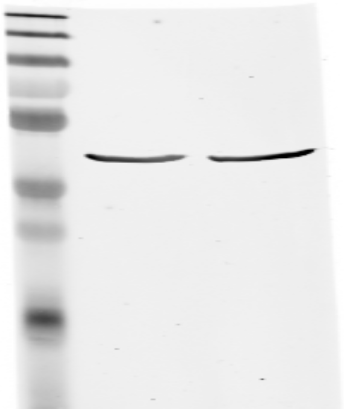

WB2: Loading control Tubulin for WB2: OCT3/4 (not quantified)

01 02

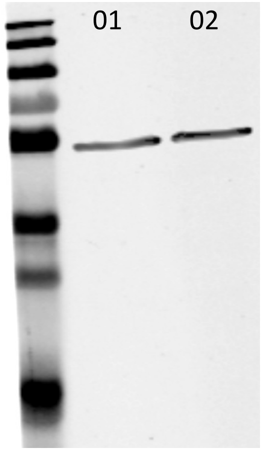

Original IP-Data: Fig. S

Tcam-2 cells: IP using antibodies directed against FLNA or FLNB and protein G beads

	5 µl pellet			25 µl pellet			10 µl sup	
FLN	B	A	M	B	A	M	B	A

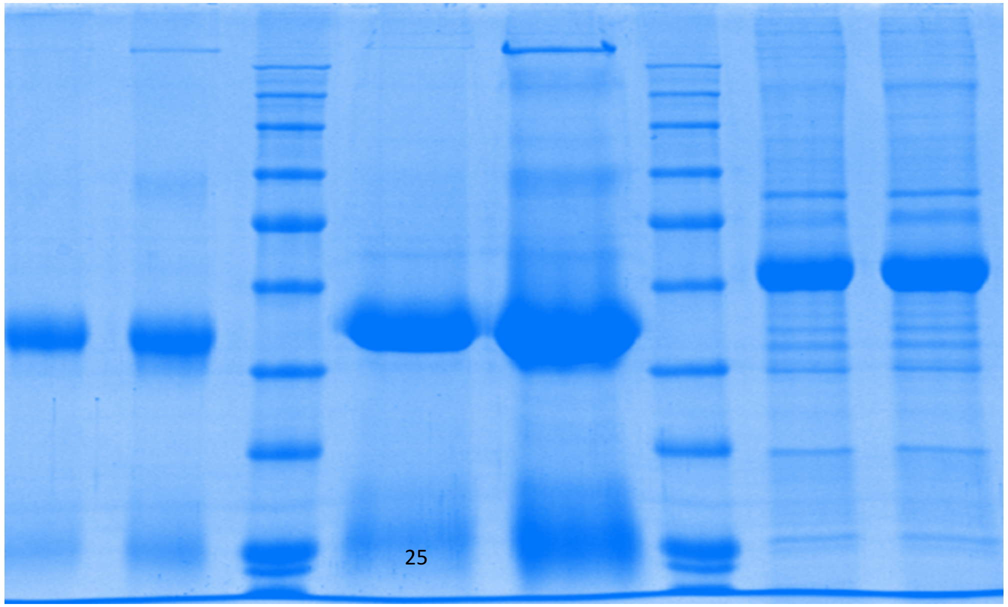

Protein G Pellet in 80 µl 2x SB > load: 5 or 25 µl

" Sup: 200 µl + 200 µl 2x SB > Load: 10 µl

Western blot analysis of potential FLN interactors

FlnB		FlnA		
S	P	S	P	M

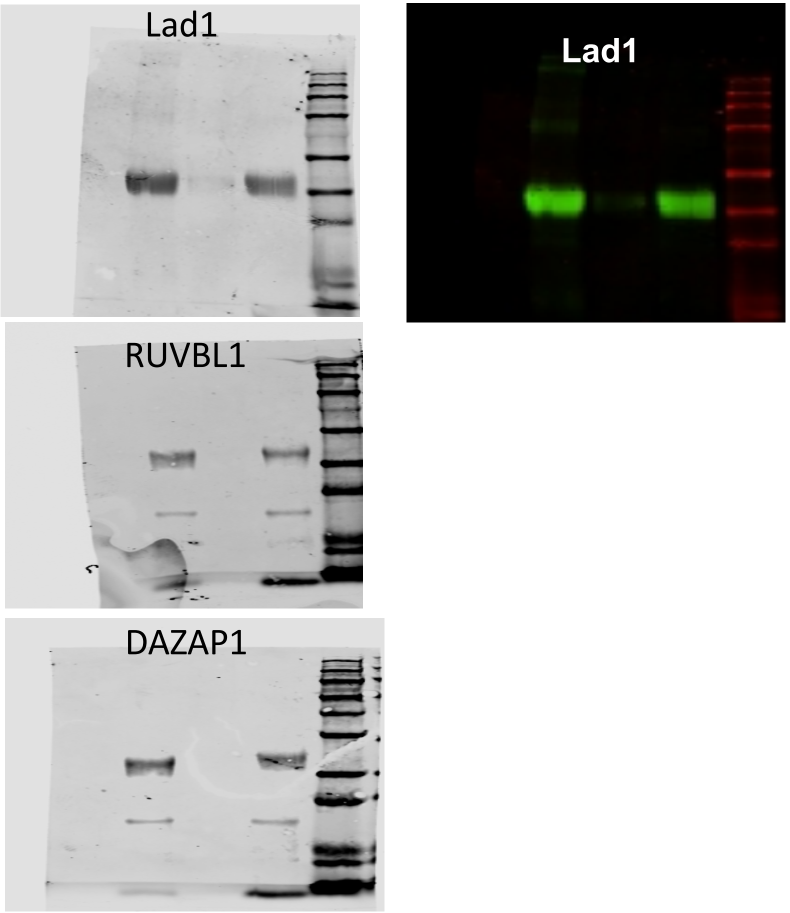

Supplement: Supplementary file 1 [file cells-09-02563-s001.zip › rev_Original lab data_cells-969341.pdf]
